# A jackknife-like method for classification and uncertainty assessment of multi-category tumor samples using gene expression information

**DOI:** 10.1186/1471-2164-11-273

**Published:** 2010-04-29

**Authors:** Wensheng Zhang, Kelly Robbins, Yupeng Wang, Keith Bertrand, Romdhane Rekaya

**Affiliations:** 1 Department of Animal and Dairy Science, University of Georgia, Athens, GA 30602, USA; 2 Department of Statistics, University of Georgia, Athens, GA 30602, USA; 3 Institute of Bioinformatics, University of Georgia, Athens, GA 30602, USA

## Abstract

**Background:**

The use of gene expression profiling for the classification of human cancer tumors has been widely investigated. Previous studies were successful in distinguishing several tumor types in binary problems. As there are over a hundred types of cancers, and potentially even more subtypes, it is essential to develop multi-category methodologies for molecular classification for any meaningful practical application.

**Results:**

A jackknife-based supervised learning method called paired-samples test algorithm (PST), coupled with a binary classification model based on linear regression, was proposed and applied to two well known and challenging datasets consisting of 14 (GCM dataset) and 9 (NC160 dataset) tumor types. The results showed that the proposed method improved the prediction accuracy of the test samples for the GCM dataset, especially when t-statistic was used in the primary feature selection. For the NCI60 dataset, the application of PST improved prediction accuracy when the numbers of used genes were relatively small (100 or 200). These improvements made the binary classification method more robust to the gene selection mechanism and the size of genes to be used. The overall prediction accuracies were competitive in comparison to the most accurate results obtained by several previous studies on the same datasets and with other methods. Furthermore, the relative confidence R(T) provided a unique insight into the sources of the uncertainty shown in the statistical classification and the potential variants within the same tumor type.

**Conclusion:**

We proposed a novel bagging method for the classification and uncertainty assessment of multi-category tumor samples using gene expression information. The strengths were demonstrated in the application to two bench datasets.

## Background

The use of gene expression profiling for the classification of human cancers has been widely investigated. Previous works were successful in predicting tumor types in the context of binary problems. Many algorithms for feature extraction and sample classification have been proposed [1-6]. More recently, a method for addressing the potential mislabeling in the training set was proposed for binary classification of cancer samples [7]. As there are over a hundred types of cancers, and potentially even more subtypes [8], it is essential to develop multi-category methodologies for molecular classification for any practical application [9].

Multi-category prediction can be achieved using binary classification algorithms via the one-versus-one (OVO) and/or one-versus-rest (OVR) partition of the training data set. However, in a cancer type prediction, multi-category problems proved to be more challenging than simple binary problems, and the reported results were less than satisfactory [3,10]. On one hand, when the available resource is limited and the sample size of a given category (class) is small, classifiers based on the OVR partition of the data set potentially suffer from severe over-fitting, leading to low predictive ability and robustness. Furthermore, the substantial noise introduced by implementing the numerous classifiers under an OVO scheme and the asymmetric training sets caused by OVR partitioning of the data will inevitably weaken the classification system. On the other hand, the effects of biological and technical noise together with the genetic heterogeneity of samples within a clinically defined tumor class decrease the predictive power in a multiple setting [11].

**Figure 1:**
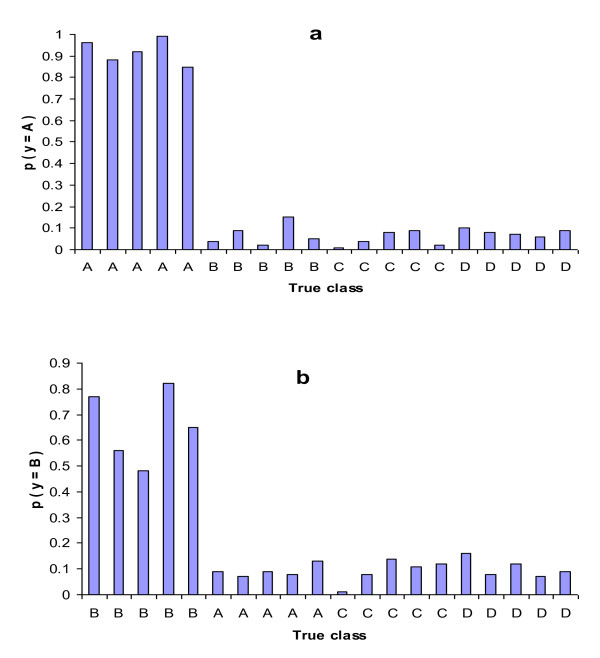
**Classification probability of a) low heterogeneity cancer type A and b) high heterogeneous cancer B using one-versus-rest (OVR) classifiers**.

In disease diagnostic, a measurement of confidence or uncertainty reported with the type determination is always desirable [6]. However, some well-established statistical criteria (such as classification probability) often become less credible and of little biological meaning for highly heterogeneous cancer types, especially in the context of multiple cancer types. A potential reason is that the winning classifier used to discriminate one cancer type from others could be weak or unstable due to limited training samples. Although this phenomenon was alluded to in previous studies [11], it has not received appropriate attention. Figure 1 presents a graphical illustration of the problem. Using an OVR binary classifier, all samples of a homogeneous cancer type (A) were classified correctly and with high confidence. All other cancer type samples in the group have probabilities of being cancer type A close to zero (Figure 1a). However, the situation was very different when a heterogeneous cancer class (B) was considered. In fact, some samples of cancer B type had classification probability lower than 0.5 (Figure 1b). Such low classification probability could lead to misdiagnosis if a hard classification rule is applied. It is possible that such low probability is due to the weakness of the classifier that is established with a highly heterogeneous training set.

The jackknife is a well known, non-parametric method often used for estimating the sampling distribution of a statistic. Given a sample dataset and a desired statistic (e.g., the mean), the jackknife works by computing the desired statistic with an element (or a group of elements) removed from the equation. The process is repeated for each element in the dataset. The application in cancer classification with gene expression profiling has been reported in the context of binary problems [12]. In that study, the individual maximum difference subsets (MDSSs) of genes identified from a set of jackknife subsets of samples were aggregated to generate the "overall MDSS" in order to return the expected classification. In other words, jackknife was used for feature selection rather than for training multiple sub-classifiers.

In this study, a new learning method called paired-samples test algorithm (PST), which is based on the jackknife method, was used to classify multiple tumor types using gene expression data. The proposed method is designed for solving multi-category problems under an OVR scheme with a very limited training data set, and it is similar to the bootstrap aggregating (bagging) procedure, which proved to be helpful in improving weak classifiers [13,14]. In order to get a relative measurement of uncertainty in the prediction of a sample category (class), the training sample being removed (validation sample) each time was predicted together with the training samples. The procedure was implemented in a parsimonious way, making its integration with a computationally intensive algorithm, such as the stochastic, regulation-based binary regression [6], feasible. The performance of the proposed method was evaluated under several scenarios of gene selection criteria using two well known and challenging datasets: the GCM and NCI60 datasets containing 14 and 9 cancer tumor types, respectively.

## Results and Discussion

Determination of the optimum number of genes (features) to be used by the classification algorithm is usually a difficult task that depends on several factors, including the classification algorithms and the complexity of the data set. For the used binary regression algorithm, previous studies have shown that a feature set of one to two hundred top genes is adequate for a simple two category problem [6,7]. In this study, the size of the feature set used was 200, 300, 500 or 1000 genes for the GCM dataset and 100, 200, 300 or 5000 genes for the NCI60 dataset.

### GCM data

**Figure 2:**
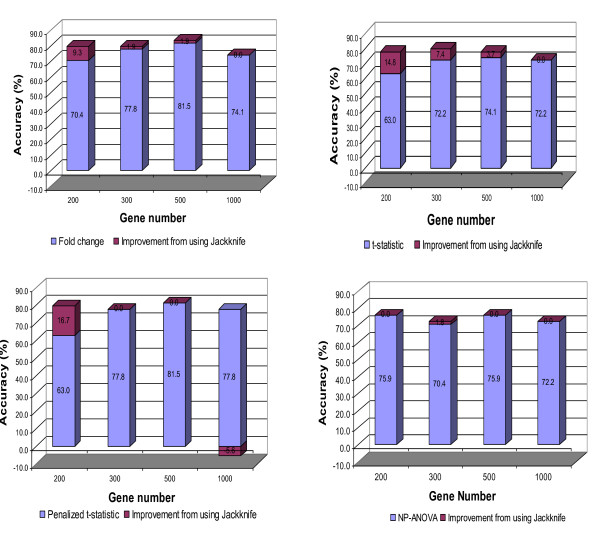
**Improvement in prediction accuracy when the proposed jackknife-like method using the independent test for GCM dataset**.

The prediction accuracy of the 54 validation samples, using different gene selection procedures, is summarized in Figure 2. The results showed that fold change and penalized t-statistic based methods for feature selection outperformed the t-statistic-based procedure. In most cases, the application of PST improved the prediction accuracy or maintained the high accuracies that had been obtained prior to its application, except in the scenario of 1000 genes and penalized t-statistic. The largest improvement occurred when 200 genes were considered using different feature selection criteria, resulting in an increase in accuracy ranging from 9.3% to 16.7%. The combination of 500 genes, fold change-based feature selection and PST had the highest prediction accuracy of 83.4%. Additionally, almost 50% of the 16.6% incorrectly classified samples had their true tumor type predicted as the second possible classification in this scenario.

It should be noted that while the largest improvements were seemingly coming from the weaker gene selection mechanisms, the application of PST made the binary regression algorithm more robust in relation to the gene selection methods and the size of the gene set to be used.

These prediction results are, in general, better than those obtained by several previous studies using the same data set (Table 1). Using a recursive feature elimination procedure and a support vector machine (SVM) classification algorithm, Ramaswamy *et al*. (2001) obtained their best result with 42 tumors correctly predicted among the 54 test samples, corresponding to an accuracy of 78% [11]. Using a feature selection algorithm based on the overlaps of gene expression values between different classes in conjunction with the Covering Classification Algorithm (CCA), a modification of the k-NN method, Bagirov *et al*. (2003) achieved a prediction accuracy of around 80% [15]. Based on the concept of gene interaction, Antonov *et al *(2004) proposed a Maximal Margin Linear Programming (MAMA) procedure that combines linear programming (LP) and SVM [16]. Using MAMA, only eight test samples were misclassified. Although slightly superior to our method (8 vs. 9 misclassified samples) in the overall accuracy, the lack of information about confusion profiles of the prediction and the secondary classification of non-correctly predicted samples make the direct comparison between both methods difficult. Recently, Sheng and Tan (2006) reported a prediction accuracy of around 83% by using error correcting out codes (ECOC), SVM and a recursive feature elimination procedure [17]. The output coding based approach is very costly in implementation and the result was highly sensitive to the decoding functions and the length of the random code.

**Table 1:** Comparison between the incorrectly classified samples obtained using the proposed method and previous studies using the same data set.

		Misclassified samples^a^		
		
Tumor Type	Test samples	Ramaswamy (2001)	Bagirov (2003)	Current Study
BR	4	2(OV, PA)	3(LU, LU, BL)	3(LE, PA, UT)
PR	6	2(OV, UT)	2(BR, UT)	
LU	4	2(BL, ML)	0	1(BL)
CO	4	0	1(UT)	1(CSN)
LY	6	0	0	0
BL	3	1(ML)	2(UT, PA)	0
ML	2	1(BL)	0	1(RE)
UT	2	0	0	0
LE	6	1(PR)	0	0
RE	3	0	2(BR, UT)	0
PA	3	1(BR)	1(CO)	0
OV	4	2(ML, PA)	0	2(BL, CL)
ME	3	0	0	1(RE)
CSN	4	0	0	0
Total	54	12	11	0
				9

^**a **^In parenthesis are the assigned tumor types for the misclassified samples.

It is possible that the superiority of the proposed method over SVM and other learning algorithms could be related to the difference in gene selection methods used in this study and by Ramaswamy *et al *(2001) and Bagirov *et al *(2003) [11,15]. However, our preliminary work as well as readily available information [10,18] demonstrated that SVM outperformed k-NN, NN (neural network), PNN (probabilistic neural work) and the decision tree in general does not support such a claim. In fact, the highest accuracies obtained using SVM occurred when 200-1000 genes were selected based on FC, t-statistics, penalized t-statistics and non-parametric ANOVA, ranging from 72.2% to 75.9%. These were well below the results obtained using our approach.

As indicated in Table 1, it seems that some tumor types are easily predicted. For example, LY, UT, ME and CSN tumors had 100% prediction accuracy using all three methods. Meanwhile, other types, such as BR, had a high misclassification rate ranging from 50 to 75%, indicating potential excess heterogeneity. Additionally, the profile of misclassified samples was very different between the four studies. In fact, among the four BR tumors, two were misclassified as OV and PA in Ramaswamy *et al *(2001) [11], three were misclassified as LU, LU and BL in Bagirov *et al *(2003) [15], and three were misclassified as LE, PA, and UT in the current study.

**Figure 3:**
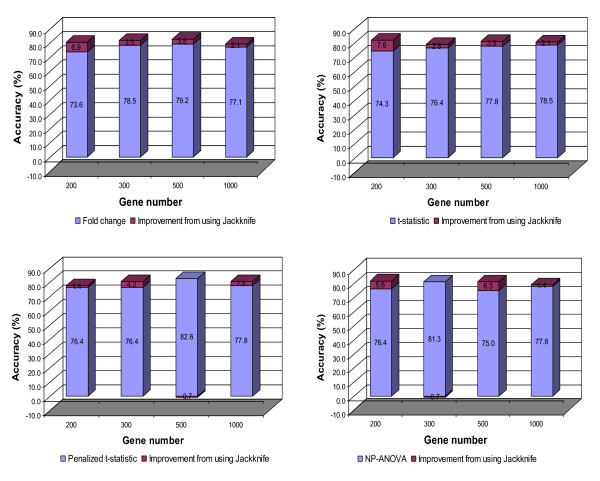
**Prediction accuracy in the cross validation for GCM dataset**.

To further validate the results behind the use of the independent 54 test samples, a four-fold cross validation was conducted for the 144 training samples. The results of this validation are presented in Figure 3. In most scenarios, the prediction accuracy was improved when the proposed Jackknife method was used. The highest value was 82.6%, which was achieved from several combinations of the gene selection method and gene number, including the case of FC-based gene selection and 500 genes. This accuracy value was similar to the best performance of 83.4% obtained using the independent 54 test samples, and it is 5.6% higher than the accuracy obtained by Ramaswamy *et al *(2001) [11].The NP-ANOVA feature selection performed marginally better in the cross validation than in the independent test with the highest prediction accuracy of 82.0%.

#### PPP rediction uncertainty

**Figure 4:**
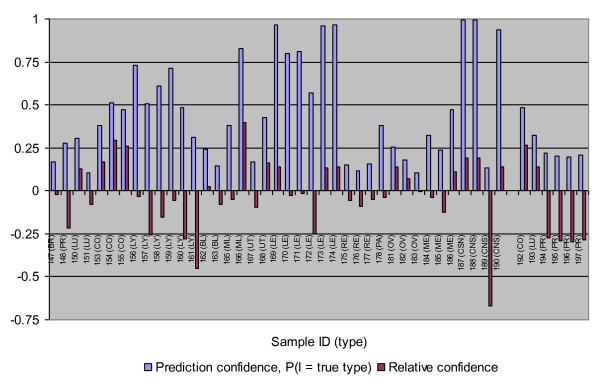
**Prediction confidence and relative confidence for the 45 correctly classified samples using fold change-based gene selection and paired-samples test algorithm in the independent test for GCM dataset**. The 6 samples to right end were metastatic tumors.

Uncertainty of the 45 correctly classified test samples from the best result is graphically presented in Figure 4. Among these 45 samples, eight tumors (1 ML, 5 LE, 3 CNS) had high *F(T) *(>.75) and nearly 3/4 of the tumors had their prediction confidence < 0.5. For the classification algorithm used in this study, *F(T) *was defined as the aggregate probability that the test sample *T *belongs to the assigned tumor type. In this context, considering *F(T) *alone makes the current prediction results seem unexpected. However, when taking *R(T) *values into account, confidence measurement, or *F(T)*, appears to be in better agreement with the results of this study. Of those samples with lower prediction confidence, the majority had their *R(T) *between -0.15 and 0.15, suggesting that their lower prediction confidences were mainly due to the potential weakness of the classifiers and/or some moderate heterogeneity. In addition, the profile of the four metastatic prostate (PR) samples was interesting. Although they were predicted with 100% accuracy, their relative confidences were low. This suggests the metastatic tumors can be distinguished from the primary tumors of the same type by using the proposed relative confidence criterion.

### NCI60 data

**Figure 5:**
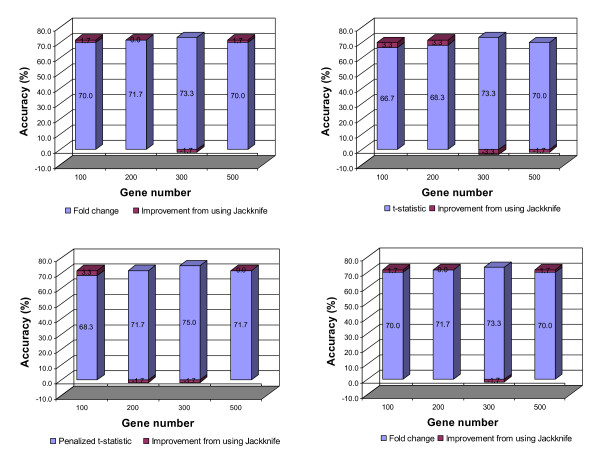
**Prediction accuracy in the cross validation for NCI60 dataset**.

There were no independent test (validation) samples in the NCI60 dataset. Consequently, ten-fold cross validation was conducted as in Statnikov et al (2005) [10]. The results are summarized in Figure 5. In most scenarios, the prediction accuracy ranged from 66.7% to 73.3%. The improvement due to the use of the PST algorithm was not as significant as with the GCM data. A modest improvement was observed when the number of used genes was relative small (200 - 300). One explanation is that, because some tumor types had a very limited number of samples (4-5 samples) available for training the classifiers, holding out one sample from the training set as is required for the implementation of PST sharpened sample shortage and weakened the trained classifiers. Nevertheless, the prediction accuracy obtained was comparable to the best reported results using this dataset. According to Statnikov et al. (2005) [10], SVM-based methods performed much better than k-NN, PNN (probabilistic neural network) and other non-SVM methods with an accuracy ranging from 47.4% to 75.0%. Furthermore, it was evident from our study that breast cancer (BR) samples were unpredictable. This result is consistent with Ross et al (2000), in which the BR samples could not be clustered together [19]. The reason could be that the BR samples contained estrogen positive (ER+) and estrogen negative (ER-) subtypes [19].

## Conclusions

In cancer type predictions, multi-category problems have proven to be more challenging than binary cases, not only in the classification accuracy but also in the assessment of uncertainty. In this paper, a jackknife-like classification method, called paired-samples test algorithm (PST), was proposed and applied to two bench datasets of multiple tumor types [11,20]. The results showed that the proposed method has improved the prediction accuracy of test samples in the GCM dataset, especially when t-statistics were used for primary feature selection. For the NCI60 dataset, improvement was observed only when the number of used genes was relative small. These improvements made the binary regression algorithm more robust to gene selection and the number of genes used.

The core idea of the proposed method is to repeatedly test a certain known tumor type with a blind test sample while withholding an associated training sample; in this way, not only can the prediction be made but also the relative confidence *R(T) *of the prediction can be accessed by measuring the difference between the prediction probability of the test sample and the corresponding value of the withheld training sample. *R(T) *provided insight into the sources of the uncertainty in the statistical classification by revealing the loss in confidence due to the utilization of weak classifiers or heterogeneity in a given tumor type. It is possible to combine the measurement *F(T) *and *R(T) *to make a better score for type determination. Our continuous work will consider this possibility in regards to penalizing a negative *R(T) *value.

## Methods

### Paired-samples test algorithm

#### Background

When the distribution of the data is complex and/or the training set is small compared to the feature dimension, the combined decision of an ensemble of multiple classifiers can be used to improve the performance of a single classification rule [13]. The bagging procedure is one such technique widely used to establish multiple classifiers [21]. It consists of training a set of classifiers, each being based on a bootstrap replicate of the training set, and aggregating them according to relative credits or weights. In the process of training classifiers for tumor prediction using microarray data, a feature selection step is usually performed in order to decrease noise. Therefore, the bagging technique could improve the robustness for the prediction mainly due to the fact that each classifier has its specific training set and group of selected features. However, for multi-category problems, the application of bagging techniques is subject to some limitations due to the partition of the training set as described in the following paragraph.

Assume the original training set includes 10 tumor types labeled with letters from *A *to *J*, and each type has 8 samples. In establishing an OVR classifier for separating type *A *from others, the training data will be divided into two groups, one containing the *A *samples (8 samples) and another containing the remaining 72 samples (B to J). Although the training set is not small in size, it is extremely asymmetric. Theoretically, in a bootstrap replicate of the same size, the probability of a sample being included is [21]. Thus, the number of *A *samples in some replicates may be very small, leading to an inaccurate classifier. Furthermore, a valid bagging technique requires a great deal of replicates. Consequently, combining a bagging technique with computationally intensive classification algorithms and gene extraction methods may become impractical due to high computational cost. In order to overcome these shortcomings, we propose a paired-samples testing algorithm, a parsimonious jackknife-like method.

#### Paired-samples test algorithm (PST)

**Figure 6:**
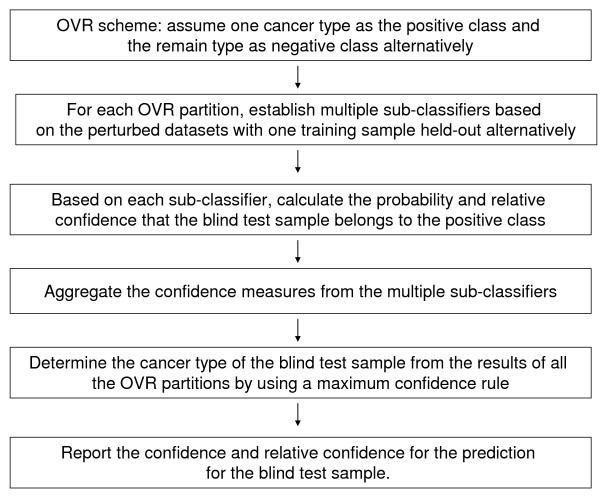
**Flow chart of paired-sample test algorithm**.

The algorithm is based on the concept of jackknifing. For every tumor type, multiple classifiers are established. The size of the classifier ensemble is equal to the number of the training samples of the tumor type in the original data. Each classifier is trained on a data set with one sample of the tumor type withheld. Using the same classifier, the withheld sample is "predicted" together with the test samples (for description convenience and without loss of generality, we assume there is only one sample in the test set). The probabilities that the two samples belong to the same tumor type are computed and a relative value is calculated as the difference of the two probabilities. By aggregating the results from the multiple classifiers, the probability that the test sample belongs to the tumor type and its relative confidence are obtained. Similarly, the same quantities (probability and relative confidence) are calculated for the remaining other tumor types in the test sample. Finally, the maximum confidence rule is used to determine the tumor type for the test sample and to report the confidence and relative confidence for the prediction. Figure 6 shows the flow process of the algorithm.

**Figure 7:**
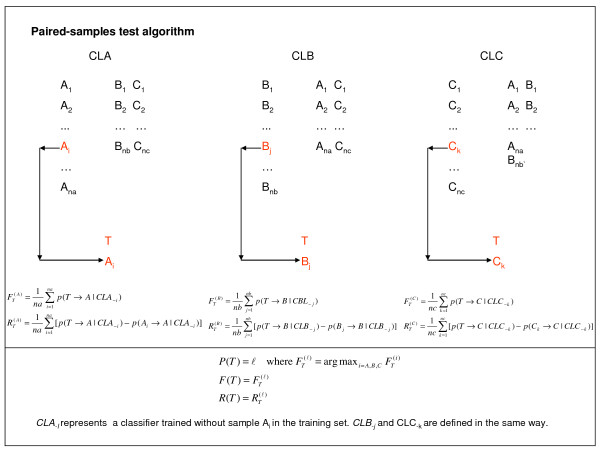
**Illustration of paired-sample test algorithm**.

The mathematical process is illustrated in Figure 7. The data set contained three tumor types labeled *A*, *B *and *C *with *na, nb *and *nc *training samples, respectively. *T *is the test sample for which the tumor type is to be determined, and CLA, CLB and CLC represent the three classifier ensembles for tumor type A, B and C, respectively. *p*(*T *→ *A*|*CLA*_- *i*_) represents the probability that test sample *T *belongs to tumor class *A *under classifier *CLA*_*-i,*_, where the subscript *i *indicates that sample A_i _was removed from the training set. All other probabilities are defined in the same way.  and  represent the confidences that *T *belongs to tumor type *A*, *B *and *C*, respectively, and  and  are the relative confidences. *P*(*T*) = λ indicates that sample *T *has been classified as tumor type λ (λ = *A*, *B *or *C*), and *F*(*T*) and *R*(*T*) are the prediction confidence and relative confidence, respectively.

#### Prediction uncertainty

**Figure 8:**
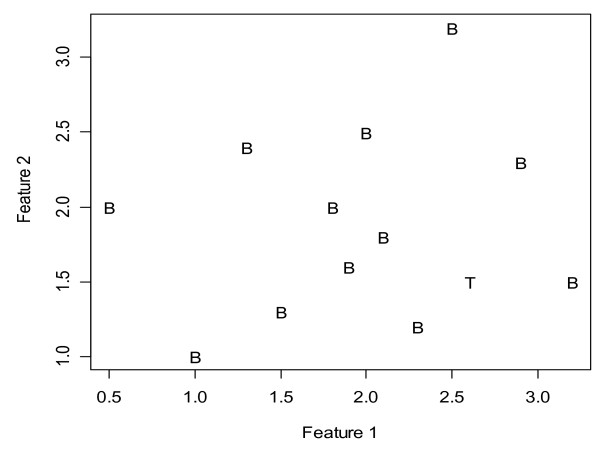
**Illustration of classification in the presence of a heterogeneous cancer type**. "B" indicates samples in training set and "T" is a test sample. The latter is closer to the center of the class than some training samples leading to a positive relative confidence.

Uncertainty is usually evaluated through its reverse relationship with some confidence measurements [6,7,11]. However, a low prediction confidence for a given sample may stem from the inadequacy of the classifiers used and/or the sample itself. In this study, for a test example T, in addition to the confidence measurement *F(T) *to be used for class determination, the relative confidence *R(T) *was calculated in order to provide insight into the sources of uncertainty in the statistical classification. It was defined as the aggregated difference between the estimates (from the series of sub-classifiers based on the training sets) of the probability that the blind test sample T belongs to the assigned tumor type, and the correspondent values for the training samples that actually belong to the class which are paired with T. As showed in Figure 1, *R(T) *measures the similarity of a particular test sample that has been assigned to a specific tumor type class relative to samples known to be of the same tumor type. If *R(T) *does not deviate much from zero, the sample could be considered as an "average" tumor of the assigned type. In this case, even a low *F(T) *does not represent a severe problem because the uncertainty is mainly due to potential limitations of the used classifier. However, when *R(T) *deviates substantially from zero in the negative direction, a small to medium magnitude for *F(T) *could indicate that the test sample is likely to be a member of an unknown subtype or variant that is absent or less represented in the training set. A large positive *R(T) *will very likely indicate a high heterogeneity between the training samples in the tumor type in which the test sample was classified as indicated in Figure 8.

### Binary classification algorithm

A binary classification algorithm was nested in PST and was performed to establish the series sub-classifiers and calculate the classification probabilities, such as *CLA*_- *i *_and *p*(*T *→ *A*|*CLA*_- *i*_) as indicated in Figure 7. Prior to each implementation, the genes were selected with the information of the involved training samples and by using the methods described in section 2.3.

In this algorithm, the relationship between the binary response, **Y **= (*Y*_1_, *Y*_2_, ... *Y*_*n*_), and the gene expression matrix, **X **= (**X**_1_, **X**_2_, .. **X**_*n*_), was modeled via a continuous and unobserved latent random variable (liability) λ = (λ_1_, λ_2_, ..., λ_*n*_) such that(1)

and(2)

where β was a vector containing model parameters. The link function of the linear predictor  with the binary response *Y*_*i *_was structured via a probit model [6,22,23]. That is(3)

where Φ (·) was the standard normal cumulative distribution function.

Due to the fact that the number of genes was larger than the number of samples, a dimension reduction technique called singular value decomposition *(SVD) *was performed on the gene expression matrix **X **[6]. The resulting model is equivalent to the one in equation (3) but with "eigen-genes" as the exploratory factors [24].The reduced regression model had the dimension of its parameter vector **γ **equal to the number of samples in the training set. The parameter vector, **γ **was estimated using Gibbs sampling, and **β **in equations (2) and (3) was obtained as a by linear transformation of the estimates of the reduced model [6,7]. The technical details of the implementation of SVD and Gibbs sampling can be found in West et al (2001) and Zhang et al (2006) [6,7]. We also tried using ICA (Independent Component Analysis) to replace SVD for dimension reduction [25], however no positive improvement in the prediction performance was achieved (results not shown).

### Feature selection

Feature selection steps were required for cancer classification with gene expression profiling. In this study, four gene ranking methods were combined with PST. All the calculations were based on log_2 _transformed gene expression data.

#### Fold change (FC)

It is calculated with the formula *FC *= |*M*_*o *_- *M*_*r*_|. In OVR setup, *M*_*o *_represents the mean of the training samples in a single tumor type to be separated from the others, and *M*_*r *_represents the mean of the training samples in all other cancer types.

#### t-statistic

It is defined as

where *S*_*p *_is the pooled standard deviation, *N*_*o *_and *N*_*r *_are the numbers of the training samples in the two groups, respectively, and *M*_*o *_and *M*_*r *_are the same as defined above. Gene ranking is based on the absolute values.

#### Penalized t-statistic

It penalizes genes with small *S*_*p *_by adding a positive quantity, *a*, to the denominator of the t-statistic, represented by the formula:

In this study, δ was set to a value equal to the ninetieth percentile of the distribution of the standard deviations of *S*_*p *_for all genes in the array [26].

#### Kruskal Wallis non-parameter test (NP-ANOWA)

The statistic in this test was defined as

where *R*_*i *_is the sum of ranks in group *i*, *n*_i _is the number of observations in the *i*^*th *^group, and n is the sample size. There are *e *distinct values, with v_1 _equal to the smallest, v_2 _equal to the next smallest and so on. In OVR setup, the test reduces to the two-sided Mann-Whitney's Test. For gene ranking, only the statistic *W *is required.

### Data

The proposed method was applied to two well-known and challenging datasets that have been analyzed previously by several groups.

#### GCM dataset

It consisted of 144 and 54 training and testing samples, respectively, representing 14 tumor types [11]. These tumor types included BR (breast adenocarcinoma), PR (prostate adenocarcinoma), LU (lung adenocarcinoma), CO (colorectal adenocarcinoma), LY (lymphoma), BL (bladder transitional cell carcinoma), ML (melanoma), UT (uterine adenocarcinoma), LE (leukemia), RE (renal cell carcinoma), PA (pancreatic adenocarcinoma), OV (ovarian adenocarcinoma), ME (pleural mesothelioma) and CNS (central nervous system). All samples were primary tumors with the exception of eight metastatic tumors in the test set. Expression data was generated using Affymetrix high-density oligonucleutide microarrays containing 16,043 known human genes or expressed sequence tags (EST). Cancer types LY, CNS and LE have more training samples in the original dataset. Based on literature [11,15] and our primary analysis of the data, samples for these three types were consistently predicted with very high accuracy (95-100%). In the current study, in order to remove the concern that the high accuracy was related to the larger training sets, only 8 training samples for each of the tumor types were used for the prediction of test samples.

#### NCI60

The 60 cell lines were derived from tumors: 8 breast, 5 central nervous system, 7 colon, 6 leukemia, 8 melanoma, 9 non-small-cell-lung-carcinoma (NSCLC), 6 ovarian, 2 prostate, and 8 renal [20]. Because of their small class size, the two prostate cell lines were excluded from our analysis. Expression data was generated using Affymetrix high-density oligonucleutide microarrays containing 6,817 human genes [20].

For both datasets, the expression intensities for each gene were calculated using Affymetrix GENECHIP analysis software. In the current study, some preprocessing was conducted on the data provided in literature [11,20]. It consisted of threshold treatment of the expression intensities with 20 for GCM data (1 for NCI60 data) and 16,000 as the lower and upper limit, respectively, after which the log_2 _transformation was applied. Further, genes with the highest transformed intensity smaller than two times the minimum expression across all samples of each dataset were deleted.

## Authors' contributions

WZ and RR developed the algorithm. WZ carried out statistical analysis and drafted the manuscript. KR, YW and KB participated in writing. All authors read and approved the final manuscript.
